# Challenges Faced by Village Health Teams (VHTs) in Amuru, Gulu and Pader Districts in Northern Uganda

**DOI:** 10.4236/ojpm.2014.49084

**Published:** 2014-09

**Authors:** Geofrey Kimbugwe, Maghanga Mshilla, Denis Oluka, Olivia Nalikka, Joseph Kyangwa, Stella Zalwango, Uthuman Kilizza, Munanura Turyasiima, Louis Ntambazi, Fred Walugembe, Julius Galiwango

**Affiliations:** 1 Faculty of Medicine, Gulu University, Gulu, Uganda; 2 Department of Medical Entrepreneurship, Faculty of Medicine, Gulu University, Gulu, Uganda; 3 Department of Pharmacology, Faculty of Medicine, Gulu University, Gulu, Uganda

**Keywords:** VHT, Gaps, Measures, Roles, Volunteer

## Abstract

Primary health care provision through innovative community level interventions such as the Village Health Team (VHT) concept in Uganda can be a rational way of achieving universal access to healthcare. This cross-sectional study interviewed 150 VHT members and 16 key informants in three districts in Northern Uganda to establish the roles of VHTs, the service gaps encountered and the measures in place to address these gaps. Quantitative data were analyzed using SPSS 16.0. Direct content analysis of themes of transcribed qualitative data was conducted manually for common codes. The majority of the respondents 64.29% (n = 72) reported to have been VHT volunteers for more than 5 years. Among the roles were community mobilization reported by 99.1% (n = 111) and home visiting of individuals reported by 97.3% (n = 109). Lack of transport, motivation, adequate skills and community appreciation with nearly no measures in place to counteract the challenges was reported by almost all respondents. Although the VHT concept can be a significant means of achieving universal access to primary health care, extensive community involvement and motivation of the volunteers are highly needed for a maximum benefit.

## 1. Introduction

The Village Health Team (VHT) concept that serves as a community’s initial point of contact for health care became part of Uganda’s National Health Strategy in 2001 [1]. Mortality and morbidity rates in Uganda are among the highest globally [2] and in response to this crisis, the Uganda Ministry of Health (MoH) created this program to bring basic preventive care to rural villages. A VHT covers the geographical size of the Local Council 1 (Village) [3] with members’ selection done on a popular vote and the team must be gender balanced with at least a third of women [4]. The very nature of Primary Health Care (PHC) necessitates community participation [5] and the origin of the VHT concept can be traced to the 1978 Alma Ata declaration, where health was entrenched as a basic human right [6] and member countries agreed that PHC through concepts like the VHTs was a means through which universal health care would be achieved and Governments were responsible for developing measures and strategies that were relevant to their unique social, economic, political and cultural conditions [7]. Studies in Latin American countries revealed that PHC had a strong potential to improve access to health care among the previously marginalized and excluded individuals [8]. Komaketch (2007) found out that the VHTs have been active in mobilization of the community for mass and routine immunizations, vitamin A supplementation and child health days as well as monitoring the maternal and neo-natal tetanus situation in their areas [9]. A Few districts meet the requirement of at least 80% of the population to be within 5 km reach of a health facility [10] and those that do face other access barriers to health especially in terms of the key health inputs like human resources, drugs and equipment [11]. Most community-based PHC groups rarely outlast their founding leaders, being often stopped by overexpansion of the population served or so many services introduced that exceed their financial and management capacity [12]. It is imperative to however note that health workers are the most powerful resource for producing good health care services which can ultimately influence the success or failure of the health system [13] [14]. A situation analysis by UNICEF (2009) concluded that VHTs can and do save lives, but their impact could be more dramatic if activities were better planned and focused on evidence-based high impact interventions [15] and hence the need for this study.

## 2. Methodology

### 2.1. Study Design

This was a cross-sectional study. Both quantitative and qualitative data were collected.

### 2.2. Study Areas

The study areas were Amuru, Gulu and Pader districts in Northern Uganda.

### 2.3. Study Duration

The study was carried out from April 2012 to April 2013.

### 2.4. Study Population

The study population included the VHT members in these communities. According to the District Health Educators’ (DHE) offices in these three study districts, Amuru had 615, Gulu had 1050 while Pader had 200 trained VHT members. This gave a total of 1865 trained VHT members as the study population at the time of conducting the study. Officials of District health departments like the District Health Educators, District Health Assistants, health facility in-charges, local leaders and community members also formed part of the study population as the key informants.

### 2.5. Sample Size

A purposive sample of 150 (one hundred and fifty) VHT members was studied. Of the total number required, the ratio for Amuru: Gulu: Pader was determined as 615:1050:200 respectively. Thus, 50 came from Amuru, 84 from Gulu and 16 from Pader districts. A total of 16 (sixteen) key informants participated in the study.

### 2.6. Sampling Procedure

Purposively selected sentinel sites at sub-county level were used as meeting points, putting into consideration the required number of respondents in each study district and also for equal representation of ideas throughout the district. Thus two sentinel sites were selected in Amuru, three in Gulu and one in Pader. In Amuru district, two health centers (HC) that is, Lamogi HC III in Lamogi sub-county and Pabbo HC III in Pabbo sub-county acted as the meeting points. At each of these health facilities, 25 (Twenty five) VHT members were mobilized with at least a representative from each of the various parishes/villages thereby giving a 100% (One hundred percent) response rate. In Gulu district, three meeting points that is Acet HC III, Te-got HC II and Bardege HC III were selected and these contributed 39 (Thirty nine), 24 (Twenty four) and 21(Twenty one) VHT members respectively, thereby giving 100% (One hundred percent) response rate. In Pader district, only one meeting point, at Pajule Trading center in Pajule sub-county was selected and a total of 16 (Sixteen) VHT members were mobilized, thus giving 100% (One hundred percent) response rate. VHT members were mobilized by the VHT leaders and their supervisors with support from the District Health Educators, District Health Assistants and the health facility in-charges with the overall process being overseen by the Principal Investigator. Of the mobilized VHT members at each meeting point, a quarter participated in the FGDs while three quarters participated in answering the questionnaires. Thus, a random sample of 13, 21 and 4 VHT members in Amuru, Gulu and Pader districts respectively participated in the FGD while the remaining 38, 63 and 12 VHT members in Amuru, Gulu and Pader districts respectively participated in filling in the questionnaires.

### 2.7. Data Collection

Data collection was carried out using a questionnaire, focused group discussions (FGDs) with a guide and key informant interviews using a guide as well. Both quantitative and qualitative data was obtained. The questionnaire was designed in English and pre-tested before the actual data collection by administering it to a total of 6 VHT members from Laroo division in Gulu district. We used two approaches in collecting the data that is the questionnaire was either self-administered in case the correspondent was fluent in English or co-administered with an interpreter in case the respondent was not conversant with English. These questionnaires had both closed and open-ended questions. The Focused Group Discussions and key informant interviews were conducted by the researchers with some asking questions and others recording and making notes from the responses given after which all the information was brought together and a final copy made per study site. The FGD and key informant guides were designed in line with the study objectives so that the elicited responses were in tandem with them.

### 2.8. Data Analysis Plan

Quantitative data was entered and analyzed using Statistical Package for Social Science 16.0 (SPSS 16.0). Direct content analysis of themes of transcribed qualitative data was conducted manually for common codes to reveal the common challenges experienced.

### 2.9. Ethical Considerations

The study followed the National Institute of Health guidelines on research involving the use of human subjects and the consent to execute the study was obtained from the Institutional Review Committee (IRC) of Gulu University, IRC clearance no GU/IRC/01/07/12. Permission to conduct the study in the three districts was obtained from each of the offices of the District Health Officers (DHOs) and the District Health Educators (DHEs) before actual data collection. Written informed consent was sought and obtained from all respondents after explaining to them the aim of the study. Confidentiality and privacy of the respondents was greatly observed. The consent form was translated into the local language to cater for those who could not understand English.

### 2.10. Study Limitations

Being a cross-sectional study, it may not necessarily give us the likely challenges in the future. Because of the limited resources, the Community knowledge, attitudes and acceptability of the VHT concept as well as the likely impacts of VHTs in the communities they serve was beyond the scope of this study.

## 3. Results

### 3.1. Social Demographics of Respondents

A total of 112 VHT members filled in questionnaires and 38 participated in focused group discussions. A total of 16 individuals were key informants. Of those who filled in questionnaires, majority, 43.8% (n = 49) were aged between 28 to 37 years, 74.1% (n = 83) were males and 24.9% (n = 29) were females. Majority 95.5% (n = 107) were peasant farmers. Majority, 73.2% (n = 82) were Catholics, 18.8% (n = 21) were Anglicans and 8.0% (n = 9) were Pentecostals. Majority, 97.3% (n = 109) had attained formal education and of these, 61.6% (n = 67) stopped in primary while 33.0% (n = 37) stopped in O-level while a significant number, 2.7% (n = 3) had no formal education. Majority, 91.1% (n = 102) were married. The summary of the respondents’ socio-demographics is shown in **Table 1** below.

### 3.2. Roles of VHT Members

In response to how respondents became part of the VHTs, majority 84.8% (n = 95) were appointed by the community members while 13.4% (n = 15) underwent a popular vote (see **Table 2**). Majority, 64.3% (n = 72) had worked for 5 or more years while 5.4% (n = 6) had been active for less than a year (see **Table 2**). Before joining the VHTs in their respective communities, 69.6% (n = 78) were community drug distributors, 37.5% (n = 42) were condom distributors, 5.4% (n = 6) were Traditional Birth Attendants (TBA) and 1.8% (n = 2) were Traditional healers/herbalists as shown in **Table 2** below. Other groups to which VHT members belonged included; parish health mobilizers, community reproductive health workers, community vaccinators, community counselors, TBA recorders and Local Council leaders.

A total of 97.3% (n = 109) reported visiting of individuals in their homes so as to check on their health statuses while 99.1% (n = 111) reported to be involved in community mobilization for health related issues. 84.8% (n = 95) reported to be carrying out health promotion and education activities, 75.9% (n = 85) reported to be involved in management of common ill health conditions while 88.4% (n = 99) reported to be involved in following up of pregnant women and post natal mothers. Neonatal follow up and recognition of danger signs was reported by 86.6% (n = 97). 80.4% (n = 90) and 77.7% (n = 87) reported to be following up discharged cases from health facilities and those on long term treatment respectively. Other reported roles included distribution of health commodities, community information management, disease surveillance, identification of health problems in the community, immunization activities, provision of basic health care messages for behavior change, children growth monitoring, community work such as school cleaning and linking of the community members to the health centers. All these were in tandem with the various roles elicited during the FGDs and Key informant interviews. The roles are summarized in **Table 3** below.

### 3.3. Gaps Encountered by VHT Members

In response to how many individuals make up a VHT, 57.1% (n = 64) reported to be in teams of less than 4 individuals while 40.2% (n = 45) reported to be in teams of 4 - 9 individuals. Majority, 82.1% (n = 92) reported to be handling 35 and more households each, a fact reinforced during FGDs and key informant interviews as respondents revealed that each individual in a VHT handles close to 100 households, with the furthest of the handled household from the VHT member’s home being within 2 - 3 km and more than 3 km as reported by 35.7 % (n = 40) and 26.8% (n = 30) respectively. Although 50.9% (n = 57) reported to be having bicycles to help them in moving to these households, 76.8% (n = 86) reportedly walk to these households. Majority, 83.0% (n = 93) reported that bicycles would be the most appropriate transport means and they advocated for each individual to receive his/her own bicycle since the Malaria Control Programme had only given one individual in each VHT.

A significant number, 47.32% (n = 53) reported that some of their fellow VHT members had lost the spirit of volunteerism and thus, they had become inactive. During one of the FGDs, a respondent stressed the fact that she was the only active individual in her team. In response to when respondents last got the basic medicines that they distribute to individuals in their communities, 52.68% (n = 59) reported that they had last received the medicines more than 6 months from the time of the study while 27.68% (n = 31) reported not to have got any basic medicines ever since recruitment. They generally acquire these medicines from health center IIs as reported by 60.7% (n = 68). When asked about the challenges faced when distributing health commodities such as insecticide treated mosquito nets and basic drugs, 73.2% (n = 82) reported that these items are not usually enough, 69.6% (n = 78) reported that these items are not usually available while 18.8% (n = 21) reported to have faced other challenges such as poor cooperation between some health workers and the VHT members. Majority, 54.5% (n = 61) reportedly lack adequate skills to deliver health care messages. Other challenges faced when delivering health care messages included lack of political support and lack of enough resources such as education materials. Challenges faced when following up patients in the communities included lack of transport means reported by 84.8% (n = 95), lack of information about the sick or those discharged from the hospitals/health facilities reported by 64.3% (n = 72) and lack of drugs to take to them as reported by 81.2% (n = 91).

In addition to these challenges, poor motivation as the government has not allocated any funds to support their activities, lack of identification tools such as IDs or uniforms and the fact that they have never received their certificates, frequent drug shortages, erratic refresher trainings, poor road network in the communities, political interference, lack of community awareness concerning VHTs since the community members expect allowances and benefits when called for meetings were among the challenges reported by the respondents.

### 3.4. Measures in Place to Overcome the Gaps

Majority, 82.1% (n = 92) had at least received a refresher training to enhance their skills while 17.9% (n = 20) reported to have not received any ever since recruitment. Respondents reported to be getting some erratic external support from several NGOs such as Amref, Bicycles Not Bombs, ABT Associate and the Anglican Diocese of Northern Uganda. Although they were promised quarterly allowances, none of the VHTs had ever received this money. They generally reported that their major motivation stems from their spirit of volunteerism as they cannot sit on the health related knowledge they obtained since individuals in their communities need it. Other forms of motivation however included T-shirts, bicycles and recognition by health workers.

## 4. Discussion of Results

### 4.1. Social Demographics of Respondents

Generally, majority, 43.75% (n = 49) were aged between 28 - 37 years, an age range for youths. This being a voluntary activity, it implies that they have to engage in other income generating activities so as to sustain themselves. This is made worse by the fact that majority, 91.1% (n = 102) were married with peasant farming reported by 95.5% (n = 107) as the source of income. The male: female ratio of 3:1 ratio is low compared to the Ministry of Health [4] recommendation for at least a third of the members to be females. A significant number, 2.7% (n = 3) had no formal education while the majority, 61.6% (n = 69) had stopped in primary. Although this translates into the fact that majority can read and write in at least a local language [4], frequent adequate refresher training to these volunteers is paramount in keeping them updated with health related information.

### 4.2. Roles of VHTs

Majority, 84.8% (n = 95) were appointed by the community members and a significant number of them, 64.3% (n = 72) had volunteered for more than 5 years (**Table 2** above). This shows how much trust the community has in these volunteers. Several preset requirements by the Ministry of Health [4] were met by the volunteers before they became part of their respective VHTs among which included being above 18 years of age, married and having prior experience in working with their respective communities for example as community drug distributors, condom distributors, traditional birth attendants or Traditional healers/herbalists (**Table 2** above). The roles executed by VHTs are in tandem with the Ugandan Ministry of Health preset roles of VHTs [3] as a strategy of establishing an innovative approach to empower communities to participate in their own health, strengthen the delivery of health services at both community and household level [4] and as a means to realize the Alma Ata declaration [8].

Home visiting of individuals is one of the major roles executed by the VHT members. They routinely go door to door on specific days of the week visiting households they are in charge of. The aims of these visits include: to find out the health statuses of individuals in these households, to deliver health education and promotion services with much emphasis on sanitation, to deliver medications as well as identifying sick individuals who are then referred to health facilities. It is through these home visits that information concerning households and individuals in the community is obtained and recorded in the health management information systems report forms. They termed these visits “community walks”.

They carry out community mobilization for health related meetings and issues such as Vitamin A supplementation, immunization activities and other health related outreaches conducted by the health facilities similar to what was reported in the Yum be district study [9]. Through these meetings, concrete solutions to the health needs of the communities are established with participation of all individuals in the villages for example by enacting laws that guide and promote sanitation in the villages they serve and it is through these laws that community members subscribe to so as to uplift the community sanitation as a whole. They also conduct health promotion and education activities for example community sensitization about proper sanitation, personal hygiene and HIV prevention. They educate individuals about the importance of having pit latrines and also participate in constructing them. They also carry out health education sessions with a focus on abstinence, being faithful and condom use in an effort to curb down the increasing HIV infections. They further encourage individuals to know their HIV statuses looking at it as a foundation in fighting the disease. They further ensure cleanliness of public places such as water bodies in order to avoid contamination and hence preventing disease out breaks.

They participate in the management of common ill health conditions and drug distribution for Neglected Tropical Diseases. These include malaria, diarrhea, sleeping sickness, trachoma, pneumonia etc. These conditions are managed within 24 hours of onset of the symptoms and in case of no improvement; they are referred to higher health facilities. VHT members are at times involved in the transfer of the patients to the health facilities in case of a severe life threatening condition or if there are signs of the care takers not willing to take such sick people to the health facilities. These health conditions are managed with drugs obtained especially from health facilities and NGOs and these drugs include Coartem, amoxicillin, Oral Rehydration Solution, Abendazole, ivermectin and paracetamol among others.

They follow up pregnant women and post natal mothers and they cited this as an obligation for them to help prevent mothers from delivering at home and reduce on the number of women attended to by unqualified personnel during labour thus contributing to a reduction in maternal morbidity and mortality. This is achieved through registering all pregnant women and keeping track of them until they deliver. They occasionally conduct village meeting with pregnant women and their husbands during which antenatal care attendance is encouraged and information concerning family planning is shared with them. During post natal follow ups, mothers are encouraged to exclusively breast feed their children up to six months and continue breast feeding up to when the child is at least 2 years of age. For neonatal follow up and recognition of danger signs, they identify danger signs such as excessive vomiting, lethargy, unconsciousness, marked diarrhea and high body temperature and in case the signs and symptoms fail to resolve within 24 hours on the medication they normally distribute or if they cannot handle such cases, these neonates are referred to health facilities. They also follow up discharged cases from the health facilities and those on long term treatment. Discharged patients are monitored by VHTs and advise them accordingly. Also, individuals on long term treatment such as those on Anti-Retroviral drugs for HIV, co-trimoxazole prophylaxis, anti-Tuberculosis drugs, among others do benefit from VHTs since they offer to them adherence visits. VHT have played a big role in achieving Directly Observed Therapy (DOT) for the Tuberculosis patients as reported by most of the health workers who were part of the key informants.

They distribute health commodities for example; condoms, insecticide treated nets and information, education and communication materials such as books and charts aimed at educating the community about behavior change and good health practices in the communities. VHTs also carryout community information management through collecting data concerning the households they are in charge of such as the number of individuals in a home, number of children below five years and the number of pregnant women which is in turn used when planning for the community. They also participate in disease surveillance and identification of health problems in the community. One of the respondents is quoted saying “We are always on the lookout for disease out breaks. For example, here in Pader where we are having the Nodding syndrome problem, we are always on the lookout for new cases so that proper care is given to them”. They keep health workers abreast with the health statuses of the communities under their jurisdiction. They further participate in immunization activities as well. One individual in each VHT is selected and trained on how to handle vaccines and how to carryout vaccination and these constitute the so called “community vaccinator”. The community vaccinators are meant to be on a look out for children below five years and ensure that these are fully vaccinated. Focus and priority is further given to children born at home since these are more likely to miss their routine immunization schedules.

Malarial control activities carried out by VHTs include clearing of bushes around homesteads and indoor residual spraying as well as encouraging people to sleep under mosquito nets. According to the latter, people found it hard to sleep under the mosquito nets as these were reported to be warm, uncomfortable or people lacked where to hang them and in some instances, they were not enough to accommodate all individuals in a given home. Indoor residual spraying has been accepted by majority of the individuals and VHT members saw it as a success. It is done by a few trained VHT members during specific periods of the year. Other roles reported by the respondents included; peer education, assessment of nutritional statuses of children, meeting with community leaders to advocate for laws that promote health statuses of the communities, de-worming children below five years of age and counseling sessions to those living with HIV/AIDs among others.

### 4.3. Gaps in VHT Activities

The VHT concept like any other previous primary health care delivery interventions in the world, has encountered numerous challenges among which include the following:

Work overload coupled with transport problems. With the number of individuals in a VHT being generally below the set standard of 9 - 10 individuals in a team [3], it was further noted that some villages had only one active individual since the other fellow volunteers had either given up due to loss of the spirit of volunteerism or some had aged and thus were too old to execute their duties. This thus translated into work overload for the active individuals. Majority of the VHT members were serving at-least 35 households against the set standard of 25 - 30, further making their work difficult. Majority of the respondents during focused group discussions confessed to be in charge of 100 and more households and yet, these are not clustered together but rather sparsely spaced. Transport problems ranged from lack of means such as bicycles and thus they have to trek while on duty to problems beyond their control such as poor roads and flooding during the rainy season. The transport problem is exacerbated by the fact that these households are so sparsely located with the majority of the VHT respondents having to move at-least 2 km from their homes to reach the furthest households they are in charge of.

Poor Motivation of the VHT volunteers could be ranked only second to the transport problem as this was reported by every individual who participated in the study. Ideally, VHT members are supposed to receive incentives such as quarterly allowances of 40,000 Ugandan shillings, exchange and tour programmes to learn from other VHTs in other parts of the country, to mention but a few. However, none of these incentives have ever been given to the VHTs in these three study districts. As such, the VHT members are left to rely on their spirit of volunteerism which in turn affects their morale and efficiency while executing their duties. Because of lack of motivation, majority of these VHT members have ended up being inactive and yet they were very interested in becoming part of these VHTs when they joined.

Lack of equipment for use in their day to day activities for example; personal protective gears, lighting equipment and equipment to aid in demonstrations such as pit latrine construction and the erratic drug supplies are among the challenges faced. Owing to the fact that majority of the VHT members walk or use bicycles to reach individuals in the community, they find it difficult to work without items like rain coats, gumboots, torches or lamps and umbrellas yet these were promised to them during recruitment. Putting into consideration the poor electricity coverage and lack of lighting means along the routes in most of the villages in northern Uganda, they reportedly find it difficult to move at night especially during emergency situation such as when a pregnant woman experiences labour pains in the wee hours of the night requiring urgent transfer to the health facility. Erratic drug supply is a problem especially when it comes to providing basic medical care to the individuals in the communities. Frequent drug shortages from health facilities have almost rendered them inactive since they cannot provide basic care during the community walks.

They reportedly experience poor interpersonal relationships especially with the politicians in the communities and some health workers. Some political leaders are not interested in their activities and those who are interested tend to interfere with them especially when it comes to issues like mobilizing community members. Health workers take them to be of a very low cadre in health care delivery. This translates into feeling of being inferior something that demotivates them.

Having inadequate skills coupled with lack of refresher trainings was another big challenge encountered. According to the VHT training manual [3], they are supposed to undergo refresher trainings and adds on modules but these have not been implemented as scheduled. As such, the few skills they acquired at the time of recruitment go on wearing out with an ultimate reduction in their effectiveness as the first level of health care delivery.

Poor housing conditions especially the lack of permanent houses and lack of communication means are among the challenges experienced. Lack of permanent houses reportedly presents problems of emigration of individuals thereby affecting the numbers of VHTs in the villages. It is further worsened by the fact that these VHTs are not considered as a priority in the government’s efforts to rebuild northern Uganda following the war through the various projects such as iron sheet distribution. “With poor housing facilities *i*.*e*. grass thatched houses, keeping of health information becomes a challenge to us as these can easily catch fire or be destroyed by heavy rains”, a statement confessed by one of the VHT members during a FGD. Lack of communications means such as mobile phones or village phones makes it difficult to send and receive information concerning the health of community members. This further makes it hard for them to coordinate activities in the villages such as outreaches, referring of patients to higher health facilities especially in emergency situations for example calling health units in case they are in need of an ambulance and or getting urgent information from the community members for example in case of a disease outbreak among others.

They reportedly lack stationery and identification gadgets. This makes it hard for them to follow up the households and also to keep up to date information relating to the health problems of individuals in these households. Other essential items like pens, information, communication and education materials for use during health education activities are reportedly not availed to them and this makes their work difficult. Identification problems coupled with lack of respect from the community members is also another setback. They have never received their identification certificates since recruitment. They also have no specific uniform which can help community members to identify them. Lack of respect from the community members to the extent of requesting for sitting allowances after health education sessions or any other activity organized by the VHTs as well as late or poor turn up for meetings has made their efforts futile.

Other challenges include: lack of proper supervision by those concerned especially at the district level, exploitation during health programmes such as during indoor residual spraying where they are used without break tea or lunch and then paid very little among others.

### 4.4. Measures in Place to Counteract the Gaps

Their primary motive is centered on the fact that they were entrusted by the community members and as such, they cannot sit on the knowledge they acquired while being trained to become VHT members. This keeps their spirit of volunteerism up since they are meant to help their communities even though they receive little or no motivation at all from the stakeholders. Majority reported that they feel like the government is no longer interested in the VHT activities owing to issues like the government’s reluctance to provide them with at least certificates and refresher trainings to enhance their skills. They reportedly hold meetings with their immediate supervisors and work with other stakeholders such as the politicians and the developmental partners. They feel more encouraged whenever they meet as a team and discuss the challenges they are facing since at most times they end up getting solutions to such challenges. In this case, most of them agreed that their supervisors and the immediate health personnel have helped them so much especially during times when they felt like quitting the programme.

They also hailed developmental partners like Amref Uganda, Bicycles Not Bombs, Anglican Diocese of Northern Uganda and ABT associate who have tried to bridge some of the gaps. These have played a crucial role in motivating them through facilitating meetings, provision of health commodities such as drugs for common ill health conditions, capacity building through refresher trainings and provision of transport refunds after the meetings. They have further tried to help them overcome the challenge of identification through provision of identification materials like T-shirts and capes although they assert that special uniforms for them would be better. They also reported that the government with other developmental partners has tried to counteract the problem of transport through provision of bicycles and this has greatly boasted the morale of those who got them. These bicycles were however given to only one member in each of the VHT with the rest left to walk while carrying out their activities. As such, this has demoralized those who never received them. Other available measures included adopting to the community needs for example poor turn up for meetings or late coming has been solved through putting more emphasis on the door to door approach where VHT members go on moving from household to household while interacting with the community members and delivering the necessary health care packages.

## 5. Conclusions and Recommendations

Village health teams have played significant roles in bridging the gap among the community, health facilities and other key developmental partners in the health sector as depicted in this study. Amuru, Gulu and Pader districts in Northern Uganda being among the districts which were mostly affected by the Lord’s Resistance Army insurgence led by Joseph Kony that ravaged the area for over two decades, implementation of the VHT concept in these areas has encountered outstanding challenges compared to other peaceful areas of the country. Thus, its implementation and realization of the goals requires extensive harmonization of the previous primary health care interventions such as the community change agents, community drug distributors and others in order to incorporate their experiences with the overall support and supervision of these volunteers by the stakeholders. Although this appears not to be the case, several strategies have been put in place to counteract these challenges and though these challenges vary amongst different communities, their impacts on the roles of the volunteers are essentially the same.

Above all, thorough community awareness, establishment of a VHT fund with pooling of all resources from the government and other stakeholders to this centralized fund in order to handle the welfare of the volunteers and a revision of the VHT concept so that the new concept is informed by this study and other previous studies, can be a rational way of maximizing benefits from the VHT model.

## Figures and Tables

**Table 1:** Respondents’ social-demographic characteristics.

	Frequency (n)	Valid Percent (%)
**Age (Years)**		
18 - 27	23	20.5
28 - 37	49	43.8
38 - 47	27	24.1
48 - 57	10	8.9
>57	3	2.7
**Total**	**112**	**100**
**Gender**		
Male	83	74.1
Female	29	25.9
**Total**	**112**	**100**
**Occupation**		
Peasant Farmer	107	95.5
Teacher	2	1.8
Small Business	2	1.8
Others	1	0.9
**Total**	**112**	**100**
**Religion**		
Anglican	21	18.8
Catholic	82	73.2
Islam	0	0
Orthodox	0	0
Pentecostal	9	8
Other	0	0
**Total**	**112**	**100**
**Education Level**		
No formal Education	3	2.7
Primary Level	69	61.6
Ordinary Level	37	33
Advanced Level	1	0.9
Certificate	2	1.8
Others	0	0
**Total**	**112**	**100**
**Marital Status**		
Single	2	1.8
Married	102	91.1
Divorced	1	0.9
Separated	1	0.9
Widowed	6	5.4
Others	0	0
**Total**	**112**	**100**

**Table 2:** How respondents became part of the VHTs, duration of working and their category of belonging before becoming VHT members.

	Frequency (n)	Valid Percent (%)
**How respondents became part of their VHTs**		
Popular vote	15	13.4
Self-appointed	2	1.8
Appointed by the community	95	84.8
**Total**	**112**	**100**
**Respondents’ duration of working as VHT volunteer**		
6months to less than 1 year	6	5.4
1 year to 2 years	8	7.1
3 years to 4 years	26	23.2
5 years and above	72	64.3
**Total**	**112**	**100**
**Respondent’s Category of belonging before becoming a VHT member**		
Community drug distributor (CDD)	78	69.6
Traditional birth attendant (TBA)	6	5.4
Traditional healer/Herbalist	2	1.8
Condom distributor	42	37.5
Others	13	11.6
**Total**	**112**	**100**

**Table 3:** Roles played by VHT members in their communities.

Role	Response n (%)	

	Yes	No
Home Visiting	109 (97.3)	3 (2.7)
Community Mobilization	111 (99.1)	1 (0.9)
Health Promotion and Education	95 (84.8)	17 (15.2)
Management of Common Ill Health Conditions	85 (75.9)	27 (24.1)
Follow Up of Pregnant Mothers	99 (88.4)	13 (11.6)
Follow Up of Post Natal Mothers	99 (88.4)	13 (11.6)
Neonatal Follow Up and Recognition of Danger Signs	97 (86.6)	15 (13.4)
Follow Up of Discharged Cases from Health Facilities	90 (80.4)	22 (19.6)
Follow Up of Cases on Long Term Treatment	87 (77.7)	25 (22.3)
Distribution of Health Commodities	86 (76.8)	26 (23.2)
Community Information Management	91 (81.2)	21 (18.8)
Disease Surveillance	85 (75.9)	27 (24.1)
Identification of Health Problems in the Community	90 (80.4)	22 (19.6)
Malaria Control Activities	96 (85.7)	16 (14.3)
Immunization	86 (76.8)	26 (23.2)
Basic Health Care Messages for Behavior Change	85 (75.9)	27 (24.1)
Others	15 (13.4)	97 (86.6)
